# Nuclear and Mitochondrial Data on *Trichuris* from *Macaca fuscata* Support Evidence of Host Specificity

**DOI:** 10.3390/life11010018

**Published:** 2020-12-31

**Authors:** Serena Cavallero, Margherita Montalbano Di Filippo, Silvia Rondón, Claudio De Liberato, Stefano D’Amelio, Klaus G. Friedrich, Federica Berrilli

**Affiliations:** 1 Department of Public Health and Infectious Diseases, Sapienza University of Rome, Piazzale Aldo Moro 5, 00185 Rome, Italy; silvia.rondon@uniroma1.it (S.R.); stefano.damelio@uniroma1.it (S.D.); 2 Department of Clinical Sciences and Translational Medicine, University of Rome Tor Vergata, Via Montpellier 1, 00133 Rome, Italy; montalbano.margherita89@gmail.com (M.M.D.F.); berrilli@uniroma2.it (F.B.); 3 Istituto Superiore di Sanità—Viale Regina Elena 299, 00161 Rome, Italy; 4 Istituto Zooprofilattico Sperimentale del Lazio e della Toscana “M. Aleandri”, Via Appia Nuova 1411, 00178 Rome, Italy; claudio.deliberato@izslt.it; 5 Fondazione Bioparco di Roma, Piazzale del Giardino Zoologico 1, 00197 Rome, Italy; klaus.friedrich@bioparco.it

**Keywords:** *Trichuris*, *Macaca fuscata*, captive animals, zoonotic risk

## Abstract

Whipworms are parasitic intestinal nematodes infecting mammals, and traditionally humans and other primates that have so far been considered infected by *Trichuris trichiura*. Recent molecular studies report a more complex scenario suggesting the presence of a species complex with several *Trichuris* taxa specifically infecting only one primate species as well as taxa able to infect a range of primate species. The systematics of the group is important for taxonomic inference, to estimate the relative zoonotic potential, and for conservation purposes. In fact, captive animals living in zoological gardens are usually infected by persistent monoxenous intestinal parasites. Here, two Japanese macaques living in the Bioparco Zoological Garden of Rome were found infected by *Trichuris* sp. Nematodes were characterized at the molecular level using nuclear (*btub* and 18S) and mitochondrial (16S and *cytb*) markers and then compared to *Trichuris* collected previously in the same location, and to other *Trichuris* infecting primates. Evidences from mitochondrial and nuclear markers allowed for the identification of *Trichuris* sp. specific to *Macaca fuscata*. Results obtained here also described a uniform taxonomic unit of *Trichuris*, separated but closely related to *Trichuris trichiura*, thus, emphasizing its zoonotic potential for workers and visitors.

## 1. Introduction

Gastrointestinal parasites infecting animals in captivity include zoonotic species and may raise public health concerns. In addition, monoxenous gastrointestinal protozoa and nematodes may cause diarrhea as a least concern or endanger non-human primate (NHP) species [1], contributing to morbidity and mortality [2]. Among the others, *Trichuris* spp. infect captive animals worldwide [3,4] and they are reported as the most prevalent species in primates living in zoological gardens in China [5].

Nematodes of the genus *Trichuris* are intestinal parasites infecting mammals including humans, with a significant degree of host affiliation [6]. Human trichuriasis, caused by the species *Trichuris trichiura*, is one of three major soil-transmitted helminthiases, affecting around 800 million people worldwide [7]. *Trichuris trichiura* was proposed to be a complex of cryptic species able to infect human and NHPs living in the wild and in captivity [8].

*Trichuris* sp. worms were found also in Italy, infecting colonies of the crab-eating macaque (*Macaca fascicularis*) used for research [9], as well as the Japanese macaque (*Macaca fuscata*) living in the Bioparco Zoological Garden of Rome [10,11]. These infections can be very persistent, due to the high environmental resistance of eggs and to the long life span of adults, therefore requiring specific treatment strategies and big economic efforts for their eradication from a colony of captive NHPs [12]. Additionally, the more recent animal welfare management approaches used in confined locations, as the setting of multiple species environments and of conditions resembling natural environments, may favor the spread and persistence of nematodes and the transmission within and among species [13]. Beside the threat for vulnerable or critically endangered animals hosted in zoological gardens, the occurrence of zoonotic parasites suggests the need for appropriate and sensitive techniques to control them and highlight the related health risks for handlers.

In this scenario, the relevance of a correct identification of these parasites is highlighted, in the attempt at defining their eventual zoonotic relevance. The present study is aimed to characterize at the molecular level *Trichuris* sp. from two Japanese macaques hosted in the Bioparco Zoological Garden of Rome, using nuclear (18S and beta-tubulin) and mitochondrial (16S and *cytb*) molecular markers. Considering the recurrent *Trichuris* infections in the Bioparco, we also compared the material collected here to previously analyzed *Trichuris* sp. from the same NHP species monitored over a decade, to estimate genetic polymorphism and phylogenetic relationship among the parasitic nematodes in the colony of Japanese macaques living in the Bioparco since the 1980s.

## 2. Materials and Methods

### 2.1. Sample Collection and Molecular Methods

Two adult Japanese macaques (*Macaca fuscata*) died at the Bioparco Zoological Garden of Rome (Italy) in January and in September of 2020. Animals were hosted in strict accordance with good animal practices and veterinary inspection procedures. The two animals belonged to a long lasting colony of Japanese macaques that were born at the Bioparco. During necropsies, adult nematodes were found in the intestinal ceca. A total of 69 worms were collected, of which 64 (21 adult males and 43 adult females) were intact, and 5 showed fragmented bodies. Nematodes were washed in saline, and morphologically identified as *Trichuris* sp. according to Jenkins (1970) [14] and Ooi et al. (1993) [15], and then fixed in 70% ethanol until molecular analyses.

A body portion was used for molecular analyses for a subset of worms, randomly selected from the two hosts. Total genomic DNA was isolated using the ISOLATE II Genomic DNA (Bioline, UK) and used for amplification of four genomic markers. Two mitochondrial regions, the ribosomal large subunit 16S (rrnLF 5′-TAAATGGCCGTCGTAACGTGACTGT-3′and rrnLR 5′-AAAGAGAATCCATTCTATCTCGCAACG-3′) and a portion of the cytochrome B *cytb* (D769 5′-GAGTAATTTTTATAATRCGRGAAGT-3′ and D770 5′-AATTTTCAGGRTCTCTRCTTCAAT-3′), and two nuclear regions, the beta-tubulin (btubF 5′-TGCTTGATGTAGTCCGCAAG-3′ and btubR 5′-GCAAAGCCAGGCATAAAGAA-3′), and the ribosomal 18S (18SF 5′-CGAACGAGACTCTGGCCTAC-3′ and 18SR 5′-CCTTGTTACGACTTTTACTTCCTC-3′) were amplified. The PCR protocols used were described by Liu et al. (2012) [16], Nissen et al. (2012) [17] and Meekums et al. (2015) [18]. All PCRs included positive and negative controls. Positive amplicons were purified using Sure Clean (Bioline, UK) and shipped to Eurofins Genomics (Germany) for sequencing.

### 2.2. Sequencing, Evolutionary Distance, and Phylogenetic Analyses

Good quality sequences were used for genetic variability estimations and comparisons with previously collected data from specimens of *Trichuris* sp. infecting in different years the Japanese Macaques living in the same premises [10,11], with the aim to infer the possible route of infection and persistence in the captive macaques of the Bioparco. In these groups of specimens, we estimated within and between group genetic distances (using the *p*-distance method). Moreover, for comparative phylogenetic purposes, *Trichuris* sp. representative of branches belonging to the evolutionary Clade 2 defined by Cavallero et al. (2019) [11] were also included. The Clade 2 includes *T. trichiura* infecting humans and other primates and other *Trichuris* spp. showing affiliation for particular NHP species. One dataset was created for each region analyzed, and alignments were tested with ModelTest (implemented in MEGA7) to compare the fit of nucleotide substitution models, according to the lowest Bayesian information criterion (BIC) score [19,20]. Phylogenetic trees were obtained using the Maximum Likelihood (ML) statistical method and 1000 bootstrap pseudoreplications using MEGA7 software [20]. For the datasets’ details, see Table 1 and Table 2.

## 3. Results

### Molecular and Phylogenetic Analyses

Thirteen good quality sequences were obtained for Dataset_16S, eleven for Dataset_cytb, twelve for Dataset_18S and twelve for Dataset_βtub. All samples showed high genetic homogeneity.

**Figure 1:**
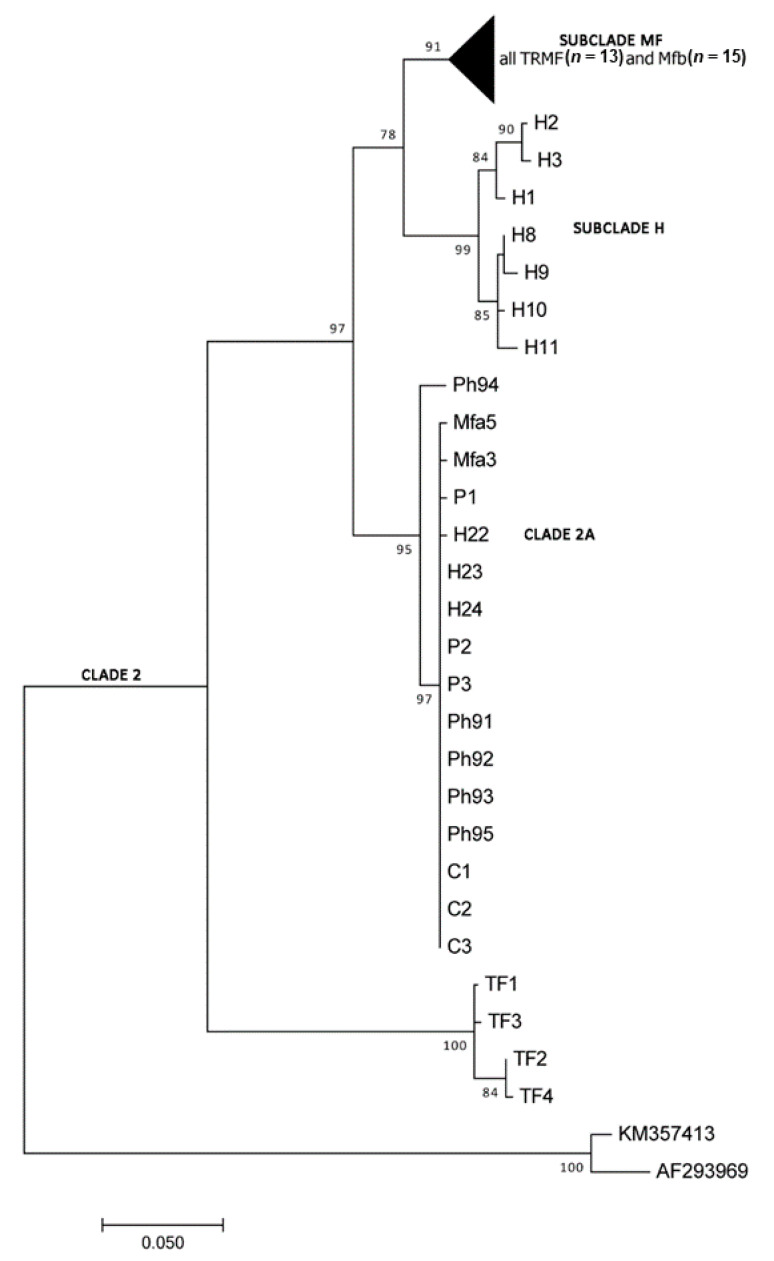
Maximum Likelihood (ML) tree elaborated from the Dataset_16S showing the evolutionary relationships of *Trichuris* spp. included (see Table 1). Bootstrap values are reported at nodes and affiliation to clades is indicated on the right.

The Dataset_16S included 50 specimens for a total length of 395 bp and the evolutionary model selected was Kimura-2 parameter + Invariant sites. The tree with the highest log-likelihood (Figure 1) described two main branches and four groups inside the Clade 2 (according to Cavallero et al. 2019 [11]). The first main branch is supported by a high bootstrap value (97%) and includes the subclade MF (bootstrap 91%) with the specimens analyzed here, and *Trichuris* from *M. fuscata* previously collected from Bioparco (Mfb specimens). The group is related with *T. trichiura* from humans (subclade H) in a branch with 78% of the statistical support. Few *Trichuris* from *M. fuscata* previously collected at the Bioparco (Mfa specimens) clustered into the Clade 2A with *Trichuris* from other primates as *Papio hamadryas*, *Chlorocebus sabaeus*, and humans (95% bootstrap). The last group was represented by the sister branch of *Trichuris* infecting *Trachypithecus francoisi*.

**Figure 2:**
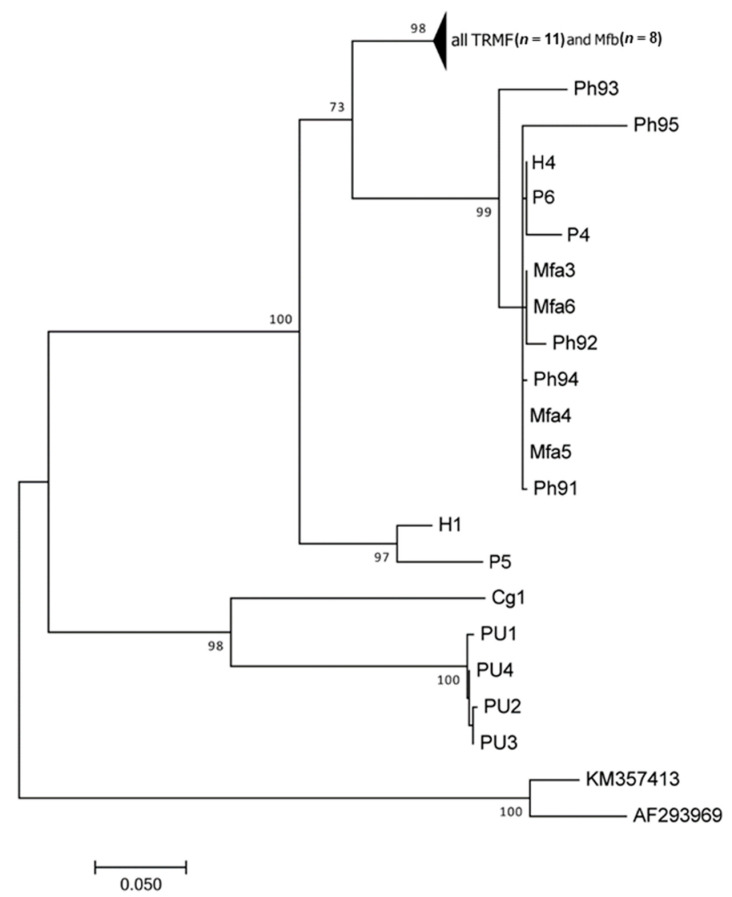
ML tree elaborated from the Dataset_cytb showing the evolutionary relationships of *Trichuris* spp. included (see Table 1). Bootstrap values are reported at nodes.

The alignment of Dataset_cytb included 40 specimens for a total length of 477 bp, with the best fit model of Kimura-2 parameter + Invariant sites. The ML tree showed the same topology described for *16S* tree, with two main branches and four groups, with slight differences in evolutionary relationships (Figure 2). The first main branch is supported by the highest bootstrap value (100%) and it includes three branches: one branch with *T. trichiura* from humans and *Trichuris* sp. from *Papio anubis* (97%), and a second branch (73%) with one group (98%) including all the specimens here analyzed and *Trichuris* from *M. fuscata* previously collected from Bioparco (Mfb specimens). A third group including *T. trichiura* from humans, *Trichuris* from *M. fuscata* previously collected at the Bioparco (Mfa specimens) and *Trichuris* from baboons. The last branch grouped *Trichuris* sp. from *Colobus guereza* and *Papio* (98%). Within mean group’s distance showed the following values: Mfa (0.001); Mfb (0.008), and TRMF (0.010). Between groups distance showed a very low *p*-distance between TRMF and Mfb (1.1%) and similar distance between TRMF/Mfa and Mfa/Mfb (13.1% and 13.3%, respectively).

The alignment of Dataset_18S included 15 specimens for a total length of 426 bp, with the best-fit model Kimura 2 parameter. All the specimens here collected form a unique clade with the two available *T. trichiura* sequences. Similarly, the alignment of Dataset_βtub included 19 specimens for a total length of 390 bp, with the best fit model Kimura 2 parameter, and showed a unique clade with all the specimens and *T. trichiura* from humans and *Trichuris* sp. from baboons. However, poor statistical support was observed at internal subclading, as reported in the consensus trees (Supplementary Figure S1).

Sequences for representative specimens for each molecular marker were submitted to GenBank (accession numbers indicated in Table 1 and Table 2).

## 4. Discussion

In this study, molecular tools were used to identify and characterize at genetic level *Trichuris* worms isolated from two Japanese macaques living in the Bioparco Zoological Garden of Rome through sequencing of nuclear and mitochondrial regions. Molecular markers as *cytb* and 16S were already used in previous analyses to characterize at the molecular level *Trichuris* sp. infecting Japanese macaques [11], while nuclear regions beta-tubulin and ribosomal subunit 18S were used to characterize specimens collected in the present work for the first time to add information on more conserved and evolutionary stable regions and to allow comparisons, according to the availability of sequences in public repositories.

The use of a molecular approach is highly recommended for species assignment, and the combination of multiple markers is useful to increase the resolution power of population genetics and phylogenetic analyses [24]. The taxonomic identity and systematics of *T. trichiura* was recently debated and several studies demonstrated the existence of more than one taxonomic entity infecting primate species, with no clear definition of species boundaries and, consequently, of zoonotic potential [8,11,23,35].

At the genetic level, all specimens collected here were very similar to each other, and one (Mfb specimens) of the two groups of previously collected *Trichuris* sp. from the same host species living in the premises in 2015–19. Specimens belonging to Mfa and Mfb were differentiated by more than fifty single nucleotide polymorphism (SNPs) at the *cox*1 analysis [13]. Based on mitochondrial markers, the level of intraspecific variation indicates the presence of a uniform population. This is even more evident in the nuclear markers, showing a limited number of SNPs (five SNPs over 390 bp in beta-tubulin and four SNPs over 426 bp in 18S region). This homogeneous taxonomic unit appears to be different from the second group (Mfa specimens) including a few specimens of *Trichuris* sp. previously collected from the same host species, more closely related to *T. trichiura* species infecting human. However, the zoonotic potential of the specimens analyzed here cannot be completely excluded. Moreover, all of these specimens belong to the Clade 2, which includes all of the putative members of the *T. trichiura* complex.

According to the last inventory of primates hosted at Bioparco (January 2020), four large areas with external and internal spaces are dedicated to stable and endangered primates. One exhibit includes orangutans (*Pongo pygmaeus*), one includes small monkeys as the emperor and the cotton-top tamarins (*Saguinus imperator* and *Saguinus oedipus*, respectively) and the pygmy marmoset (*Callithrix pygmaea*), another is for the chimpanzees (*Pan troglodytes*), and the last one includes the ring tailed lemurs (*Lemur catta*). Additional small areas host the collared mangabeys (*Cercocebus torquatus*), the capuchin monkeys (*Sapajus* sp.) and the mandrill (*Mandrillus sphinx*). With the exception of orangutans and chimpanzees, all the other primates’ premises are in the same side of the Bioparco. The distribution of premises should be accounted for a proper evaluation of transmission patterns of pathogens. In fact, the possibility of events of cross infection between premises and primates were already reported in the Bioparco between *Lemur catta* and *Macaca fuscata* [13].

*Trichuris* has been observed to infect *M. fuscata* hosted in the Bioparco for over a decade. Besides the pens’ flooring and the structure of the exhibit, a possible explanation for the persistence of such infections in the premises can be related also to the administration of pharmacological therapies with food, given the context of hierarchical competition. In fact, access to food could be influenced by social dynamics of primates, and the dominant animals may eat more food than others, thus limiting the efficacy of pharmacological treatments.

In conclusion, this study gives further evidence that, although more than one *Trichuris* sp. taxon is able to infect primates, a strong host affiliation/specificity appears characteristic of specimens infecting *M. fuscata*. However, as the number of specimens analyzed from captive primates, including Japanese macaques, are still scarce and this is even poorer from wild specimens, the present results highlight the need for further data to infer the taxonomy of *Trichuris* sp. from primates with due accuracy.

## Figures and Tables

**Table 1:** Materials analyzed in the present study for phylogenetic inferences based on two mitochondrial markers (16S and *cytb*). In bold is the material analyzed here.

Parasite Species	Host Species	GenBank Accession Number	Specimen Code	Authors and References
**Dataset_16S**				
*T. trichiura*	*Homo sapiens*	GU385218 AM993017-18	H1 H2-3	Liu et al. (2012) [16]
*T. trichiura*	*Homo sapiens*	KP781898-KP781901	H8–11	Meekums et al. (2015) [10]
*T. trichiura*	*Homo sapiens*	KU524541-43	H22–24	Hawash et al. (2016) [21]
*Trichuris* sp.	*Macaca fuscata*	MW403712-16	TRMF4,34,48,61,72 plus 8 undeposited *	Present study (representative specimens)
*Trichuris* sp.	*Macaca fuscata*	MN088542-43MN088544-58	Mfa3,5 Mfb2–4,6–8, 10–14,16–19	Cavallero et al. (2019) [11]
*Trichuris* sp.	*Papio sp.*	KU524558-60	P1–3	Hawash et al. (2016) [21]
*Trichuris* sp.	*Papio hamadryas*	MN088578-82	Ph92–96	Cavallero et al. (2019) [11]
*Trichuris* sp.	*Chlorocebus sabaeus*	KU524595-97	C1–3	Hawash et al. (2016) [21]
*Trichuris* sp.	*Trachypithecus francoisi*	KC481232-35	TF 1–4	Liu et al. (2013) [22]
**Dataset_cytb**				
*T. trichiura*	*Homo sapiens*	GU385218	H1	Liu et al. (2012) [16]
*T. trichiura*	*Homo sapiens*	KT449826	H4	Hawash et al. (2015) [23]
*T. colobae*	*Colobus guereza*	LM994704	Cg1	Callejón et al. (2015) [24]
*Trichuris* sp.	*Macaca fuscata*	MW403707-11	TRMF4,34,44,58,61 plus 6 undeposited	Present study (representative specimens)
*Trichuris* sp.	*Macaca fuscata*	MK914550-53MK914554-61	Mfa3–5 Mfb2–4,7,10–13	Cavallero et al. (2019) [11]
*T. ursinus*	*Papio ursinus*	LT627357-60	PU1–4	Rivero et al. (2020) [25]
*Trichuris* sp.	*Papio hamadryas*	KT449824	P4	Hawash et al. (2015) [23]
*Trichuris* sp.	*Papio hamadryas*	MK914573-77	Ph91–95	Cavallero et al. (2019) [11]
*Trichuris* sp.	*Papio anubis*	KT449825	P5	Hawash et al. (2015) [23]
*Trichuris* sp.	*Papio* sp.	LM994703	P6	Callejón et al. (2015) [24]
**Outgroup species**				
*Trichinella britovi*		KM357413		Mohandas et al. (2014) [26]
*Trichinella spiralis*		AF293969		Lavrov and Brown (2001) [27]

* each specimen is labeled according to TRMF code as for TR = *Trichuris* and MF = *Macaca fuscata*, followed by the number of isolate.

**Table 2:** Materials analyzed in the present study for phylogenetic inferences based on two nuclear markers (*beta tubulin* and ribosomal 18S). In bold is the material analyzed here.

Parasite Species	Host Species	GenBank Accession Number	Specimen Code	Authors
**Dataset_βtub**				
*T. trichiura*	*Homo sapiens*	AF034219	H4	Bennett et al. (1999) [28]
*T. trichiura*	*Homo sapiens*	KF410623-24	H5-H6	Hansen et al. (Unpublished) [29]
*Trichuris* sp.	*Papio hamadryas*	KF410632-34	P7-P9	Hansen et al. (Unpublished) [29]
*Trichuris* sp.	*Macaca fuscata*	MW403705-06	TRMF4,48 plus 10 undeposited *	Present study (representative specimens)
**Outgroup species**				
*Trichinella spiralis*	*Rattus norvegicus*	XM_003369432	Tspi	Mitreva et al. (2011) [30]
**Dataset_18S**				
*T. trichiura*	*Macaca fuscata*	AB699092	TtMF1	Arizono et al. (2012) [31]
*Trichuris* sp.	*Macaca fuscata*	MW396470-71	TRMF4,6 plus 10 undeposited *	Present study (representative specimens)
*T. trichiura*	*Homo sapiens*	AB699090	TtHS1	Arizono et al. (2012) [31]
*T. trichiura*	*Homo sapiens*	GQ352553	TH1	Putaporntip et al. (2010) [32]
*T. trichiura*	*Homo sapiens*	MF288632	TZY	Phosuk et al. (2017) [33]
**Outgroup species**				
*Trichinella spiralis*	*Sus scrofa*	AY497012	Tspi	Li et al. (Unpublished) [34]

* each specimen is labeled according to TRMF code as for TR = *Trichuris* and MF = *Macaca fuscata*, followed by the number of isolate.

## Supplementary Materials

The following are available online at https://www.mdpi.com/2075-1729/11/1/18/s1, Figure S1: ML trees elaborated from the Dataset_18S (a) and the Dataset_btub (b) showing the evolutionary relationships of *Trichuris* spp. included (see Table 2). Bootstrap values are reported at nodes.
