# Supplementary material for: Nuclear and Mitochondrial Data on Trichuris from Macaca fuscata Support Evidence of Host Specificity
Source: Life (Basel). 2020 Dec 31;11(1):18. doi: 10.3390/life11010018 (PMC7823418; doi:10.3390/life11010018)
Supplement: Supplementary file 1 [file life-11-00018-s001.pdf]

Supplementary Materials of Nuclear and Mitochondrial Data on *Trichuris* from *Macaca fuscata* Support Evidence of Host Specificity

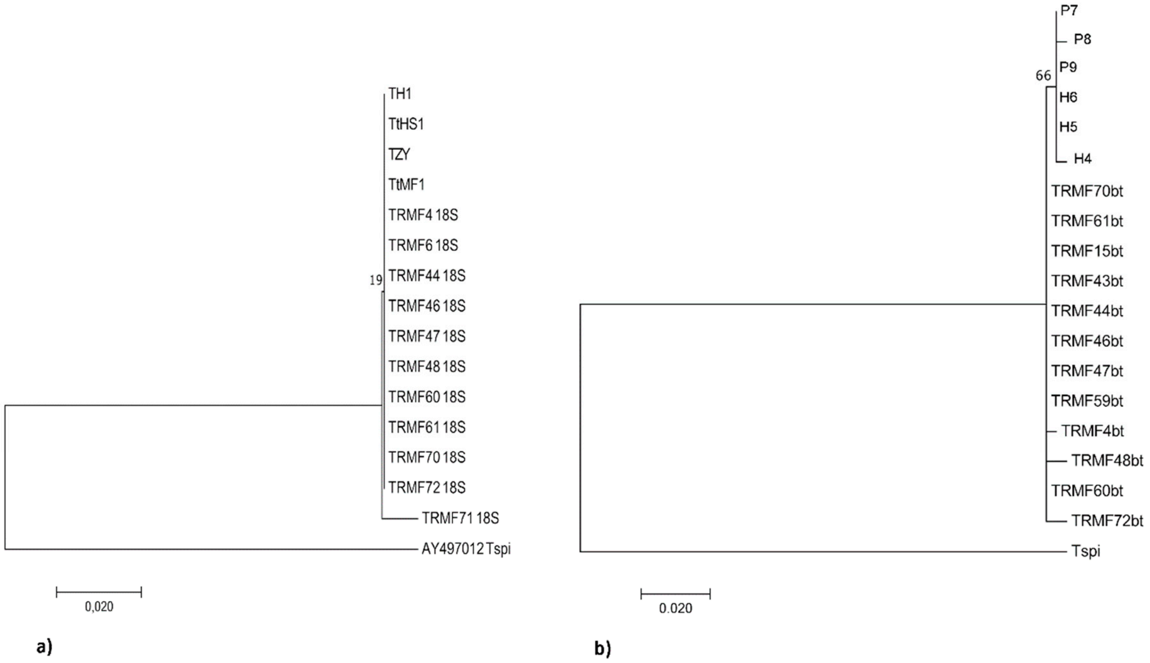

Figure S1. ML trees elaborated from the Dataset_18S (**a**) and the Dataset_btub (**b**) showing the evolutionary relationships of *Trichuris* spp. included (see Table 2). Bootstrap values are reported at nodes.
